# The role of neoadjuvant chemotherapy followed by interval debulking surgery in advanced ovarian cancer: a systematic review and meta-analysis of randomized controlled trials and observational studies

**DOI:** 10.18632/oncotarget.23808

**Published:** 2017-12-27

**Authors:** Meng Qin, Ying Jin, Li Ma, Yan-Yan Zhang, Ling-Ya Pan

**Affiliations:** ^1^ Department of Obstetrics and Gynecology, Peking Union Medical College Hospital, Chinese Academy of Medical Sciences and Peking Union Medical College, Beijing 100730, China; ^2^ Department of Interventional Radiology and Vascular Surgery, Taiyuan Center Hospital, Taiyuan 030009, China

**Keywords:** neoadjuvant chemotherapy, debulking surgery, ovarian cancer, survival

## Abstract

**Objective:**

We aimed to performed a meta-analysis and systematic review on the role of neoadjuvant chemotherapy followed by interval debulking surgery (NACT-IDS) in advanced ovarian cancer (AOC) patients.

**Materials and Methods:**

We searched PubMed, EMBASE, and the Cochrane Library for relevant articles. All statistical analyses were performed in Review Manager 5.3.5.

**Results:**

In two randomized controlled trials (RCTs), there was no significant difference in overall survival (OS) (HR = 0.93, 95% CI: 0.81–1.06) or progression-free survival (PFS) (HR = 0.97, 95% CI: 0.86–1.09). Few adverse events (HR = 0.37, 95% CI: 0.19–0.72) and a high optimal debulking surgery rate (HR = 1.69, 95% CI: 1.50–1.91) were observed with NACT. In 22 observational studies, primary debulking surgery (PDS) yielded better OS (HR = 1.38, 95% CI: 1.19–1.60) but not progression-free survival (PFS) (HR = 1.03, 95% CI: 0.86–1.23). An increased optimal cytoreduction rate (HR = 1.17, 95% CI: 1.12–1.22) was observed with NACT. Irrespective of the degree of residual disease, OS was longer in the PDS group than that in the NACT group. Patients with FIGO stage III (HR = 1.43, 95% CI: 1.05–1.95) and IV (HR = 1.14, 95% CI: 1.06–1.23) disease had better survival with PDS.

**Conclusions:**

Treatment with NACT-IDS improves perioperative outcomes and optimal cytoreduction rates, but it may not improve OS. NACT-IDS is not inferior to PDS-CT in terms of survival outcomes in selected AOC patients. Future studies should focus on candidate selection for NACT.

## INTRODUCTION

Epithelial ovarian cancer is currently the most malignant gynecological carcinoma worldwide. Approximately two-thirds of patients are initially diagnosed at an advanced stage, which is largely due to the lack of specific symptoms or early detection methods for ovarian cancer [1, 2]. Primary debulking surgery (PDS) followed by platinum-based chemotherapy has been the standard treatment for patients with advanced ovarian cancer (AOC) for a long time [3]. Optimal debulking surgery has been defined as surgery that results in macroscopic residual disease of less than 1 cm in maximum tumor diameter, and it leads to much better survival in patients than does suboptimal debulking surgery [4].

The management of ovarian cancer has changed during the past few decades because not all patients are candidates for PDS followed by chemotherapy (PDS-CT), due to either extensive tumor burden or poor performance [5]. In this situation, neoadjuvant chemotherapy (NACT) followed by interval debulking surgery (IDS) is an alternative treatment option for patients who are initially unlikely to undergo optimal debulking surgery, particularly patients with FIGO stage IIIC or IV disease. NACT refers to chemotherapy that is administered to reduce the tumor burden before debulking surgery is performed [6]. The definition of NACT-IDS has changed during the past two decades, and there are two different definitions for this term [4]. One definition corresponds to a second attempt at cytoreduction after a suboptimal initial surgical attempt [7], which is commonly referred to as secondary cytoreduction [8–10]. The second definition is currently widely acknowledged by gynecologic oncologists and considers NACT as the primary treatment, thus viewing the following surgery as interval cytoreduction [11]. It should be emphasized that only the second definition of NACT-IDS will be discussed in our article.

Recently, two multicultural, randomized phase III trials (EORTC 55971 [12] and CHORUS [13]) have reported that NACT is not inferior to primary surgery, and this has led to extensive concern [14, 15]. This conclusion has been reported in several retrospective cohort studies [16] and questionnaires [17] by gynecologic oncologists. However, the role of NACT remains controversial because it has not yet been shown to be better than PDS in promoting survival [12, 18]. The overall survival (OS) in the NACT groups in these two articles was significantly shorter than that in other randomized controlled trials (RCTs) [19]. Thus, it is still unknown whether patients who achieve microscopic residual disease with NACT have an equally good prognosis and survival as do patients who undergo PDS. Experienced gynecologists should thus conduct a complete assessment before administering NACT.

Therefore, we aimed to perform a meta-analysis and systematic review of the role of NACT-IDS in AOC patients. The OS, progression-free survival (PFS), optimal debulking rate, adverse events, and characteristics related to OS were evaluated.

## MATERIALS AND METHODS

We conducted this study according to the Preferred Reporting Items for Systematic Reviews and Meta-Analyses (PRISMA) checklist.

### Literature search

We searched PubMed, EMBASE, and the Cochrane Library for all articles until December 31, 2016 using combinations of the following keywords: “ovarian cancer”, “neoadjuvant chemotherapy”, and “debulking surgery”. The specific search strategy was the following: ((((((ovarian cancer) OR ovarian carcinoma) OR ovarian neoplasms) OR ovarian tumor)) AND ((neoadjuvant chemotherapy) OR preoperative chemotherapy)) AND ((((cytoreductive surgery) OR debulking surgery) OR interval debulking surgery) OR interval cytoreduction).

We also searched clinical trial registries, scientific conference summaries, and the reference lists of relevant studies. Articles without full text, studies lacking the outcomes of interest, and duplicate publications or overlapping populations were excluded. Among the repeated studies with overlapping patient populations, data from the study with the largest sample size and from the more recent publications were included in the meta-analysis.

### Study selection

Eligible studies were included if they met the following Patient, Intervention, Comparison, Outcomes, and Setting (PICOS) criteria: (1) P—Participants/patients: The study included women with clinical or imaging evidence of a pelvic mass compatible with FIGO stage III or IV ovarian, fallopian tube, or primary peritoneal cancer. (2) I—Intervention: The study included the intervention NACT-IDS in one of the groups. (3) C—Comparisons: The study included the comparison of NACT versus PDS. (4) O—Outcomes: The study included at least one of the outcomes of interest such as OS, PFS, or survival of the subgroups. OS was defined as the period from randomization until death from all causes using data for sur*vivo*rs from the time at which they were last known to be alive by the researchers. PFS was equated with disease-free survival and was defined as the time from randomization until the date of first progression or death from all causes, whichever occurred first. (5) S—Study: Only studies published in English were included. The type of study design was not restricted; both RCTs and observational studies were included. Studies were included in the RCT group if they were RCTs and fulfilled criteria 2 and 3, and the other studies were included in the observational studies group.

### Data extraction

Two authors independently reviewed all searched studies and extracted critical data using a predefined standard format. A third reviewer was involved in a discussion to resolve differences. The following data were extracted from each eligible study: study ID (last name of first author and year of publication), study design, country, recruitment period, FIGO stage of disease, and chemotherapy regimen. Meanwhile, the number of patients, median age (years), median OS (months), median PFS (months), and optimal debulking rates were recorded for the NACT and PDS groups. The percentage of patients who had undergone NACT followed by IDS among all patients who underwent NACT irrespective of IDS is also presented in the table. All extracted data were included in an intention-to-treat (ITT) analysis as much as possible, i.e., participants were evaluated when they were enrolled.

### Quality assessment

Two reviewers independently assessed the risk of bias for RCTs in accordance with the guidelines in the Cochrane Handbook for Systematic Reviews of Interventions in Chapter 8 (Higgins 2011)[20]. The Cochrane Collaboration’s tool has seven domains of bias: (1) random sequence generation (selection bias), (2) allocation concealment (selection bias), (3) blinding of participants and caregivers (performance bias), (4) blinding of outcome assessment (detection bias), (5) incomplete outcome data (attrition bias), (6) selective outcome reporting (reporting bias), and (7) other bias. There are specific and clear instructions in this tool to help reviewers assess the risk of bias as “high”, “low”, or “unclear”.

For observational studies, the quality of the included studies was assessed using the Modified Newcastle-Ottawa Score [21], which allocates a maximum of 9 points each to patient selection, the comparability of the two groups (NACT and PDS), and outcome assessment. Two reviewers independently evaluated the quality of the included observational studies based on detailed data. A third investigator helped resolve any disagreements for all studies.

### Statistical analysis

All statistical analyses were performed using Review Manager 5.3.5 for Mac (provided by the Cochrane Library). We performed a meta-analysis of OS and PFS using a conventional inverse variance statistical method, with the hazard ratio (HR) and corresponding 95% confidence intervals (CIs), which were either directly extracted from the studies or calculated using Kaplan-Meier survival curves from the selected studies [22]. Adverse events were analyzed to evaluate the safety of treatment. Subgroup analyses using the Mantel-Haenszel statistical method were performed in our meta-analysis, including analyses based on the extent of debulking surgery, degree of residual disease and FIGO stage, using risk ratio (RR) values and corresponding 95% CIs.

Additionally, the I^2^ statistic was used to quantify the degree of heterogeneity, which represents the percentage of overall variability across studies due to heterogeneity. I^2^ values of 25%, 50% and 75% corresponded to low, moderate and high degrees of heterogeneity, respectively [23]. Generally, *P*-values < 0.1 or I^2^ > 50% indicated significant statistical heterogeneity among the studies, and a random-effects model was thus used; otherwise, a fixed-effects model was used [24, 25].

A sensitivity analysis was performed to assess the stability of the results and to reduce heterogeneity by removing each trial one by one. A sensitivity analysis was conducted in the following situations: 1) trials with high and unclear risk of bias versus trials with low risk of bias and 2) trials with significant heterogeneity or trials differing from others in their clinical criteria [26].

## RESULTS

### Study selection

**Figure 1:**
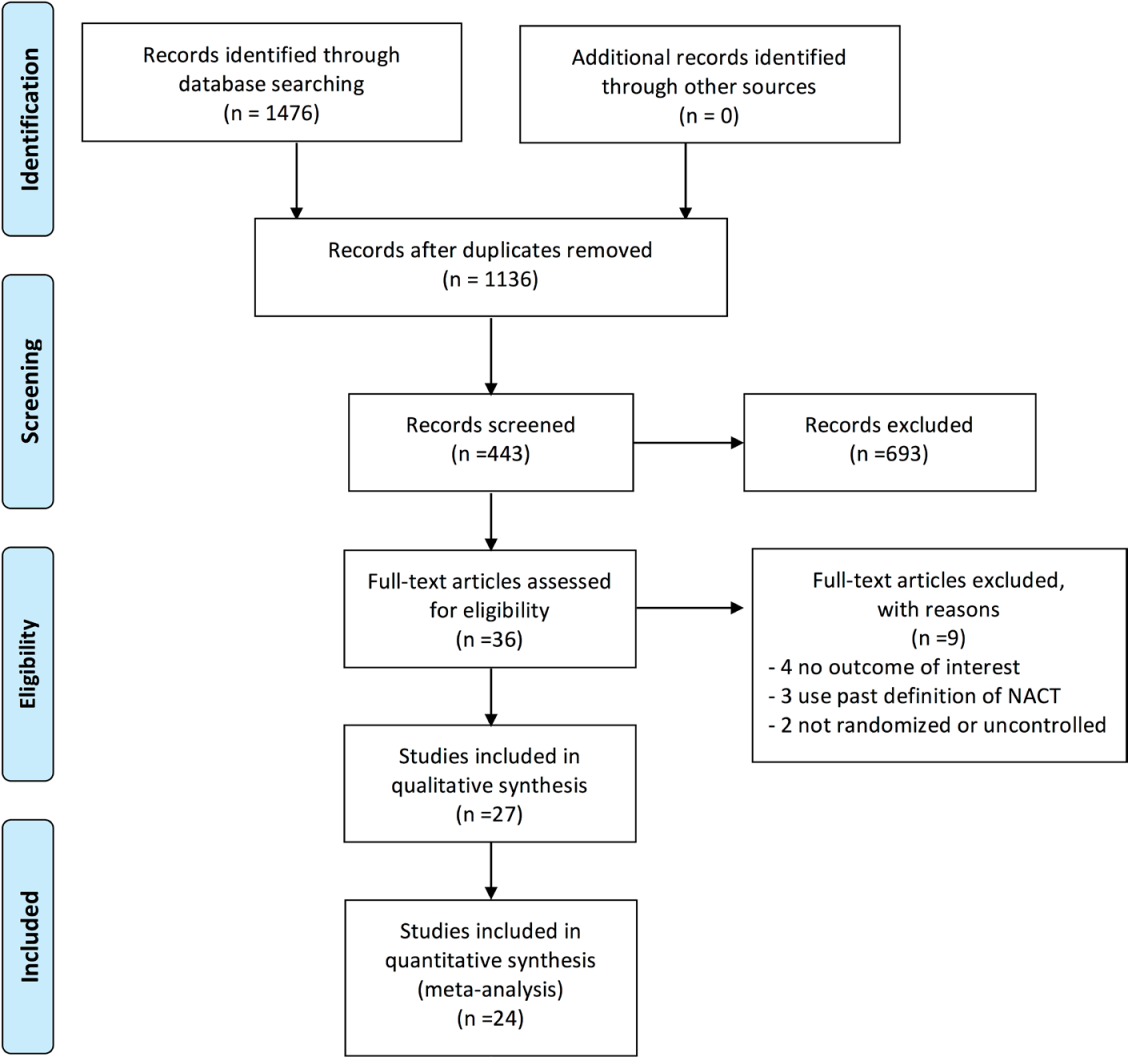
Flow diagram of trial selection

Figure 1 shows the results of article selection. We identified 1476 records from the previously listed databases; 1136 papers remained after duplicates were removed, and 36 full-text articles were assessed for eligibility. Among these 36 trials, four trials [27–30] had no outcome of interest, three trials [8–10] used former and improper definitions of NACT-IDS, and two trials [31, 32] were not randomized or controlled. Then, 27 studies [12, 13, 16, 33–57] were included in the qualitative analysis. Finally, a total of 24 studies [12, 13, 16, 37–57] from the quantitative analysis were included in our meta-analysis.

### Study characteristics

The main characteristics of these 24 studies are listed in Supplementary Table 1. There were two RCTs [12, 13] and 22 observational studies, which consisted of 21 retrospective cohort studies [16, 37–54, 56, 57] and one retrospective case-control study [55]. Because all observational studies were retrospective in nature, we also called this group the retrospective study group. Most of the trials were conducted in Europe. All studies had a rather long recruitment period and patients with a median age from 51 to 75 years. Most patients in the PDS groups were treated with first-line chemotherapy for 6 cycles after primary surgery. Similarly, most patients in the NACT group received 3 cycles of chemotherapy followed by IDS and then 3 cycles of adjuvant chemotherapy. The chemotherapy regimens were cisplatin or carboplatin combined with paclitaxel or cyclophosphamide, and the regimens did not significantly differ between adjuvant chemotherapy and neoadjuvant chemotherapy.

A total of two RCTs with 1220 patients were analyzed; 608 patients were treated with NACT-IDS, and 612 patients were treated with PDS-CT. The OS, PFS, and optimal debulking rates of the NACT group were better than those of the PDS group in both trials. In the observational studies group, a total of 22 retrospective studies including 12,775 patients were analyzed; 5,299 patients were in the NACT group, and 7,476 patients were in the PDS group. The OS and PFS in the PDS group were significantly longer than those in the NACT group. The optimal debulking rate was better in the NACT group than that in the PDS group.

### Quality assessment

**Figure 2:**
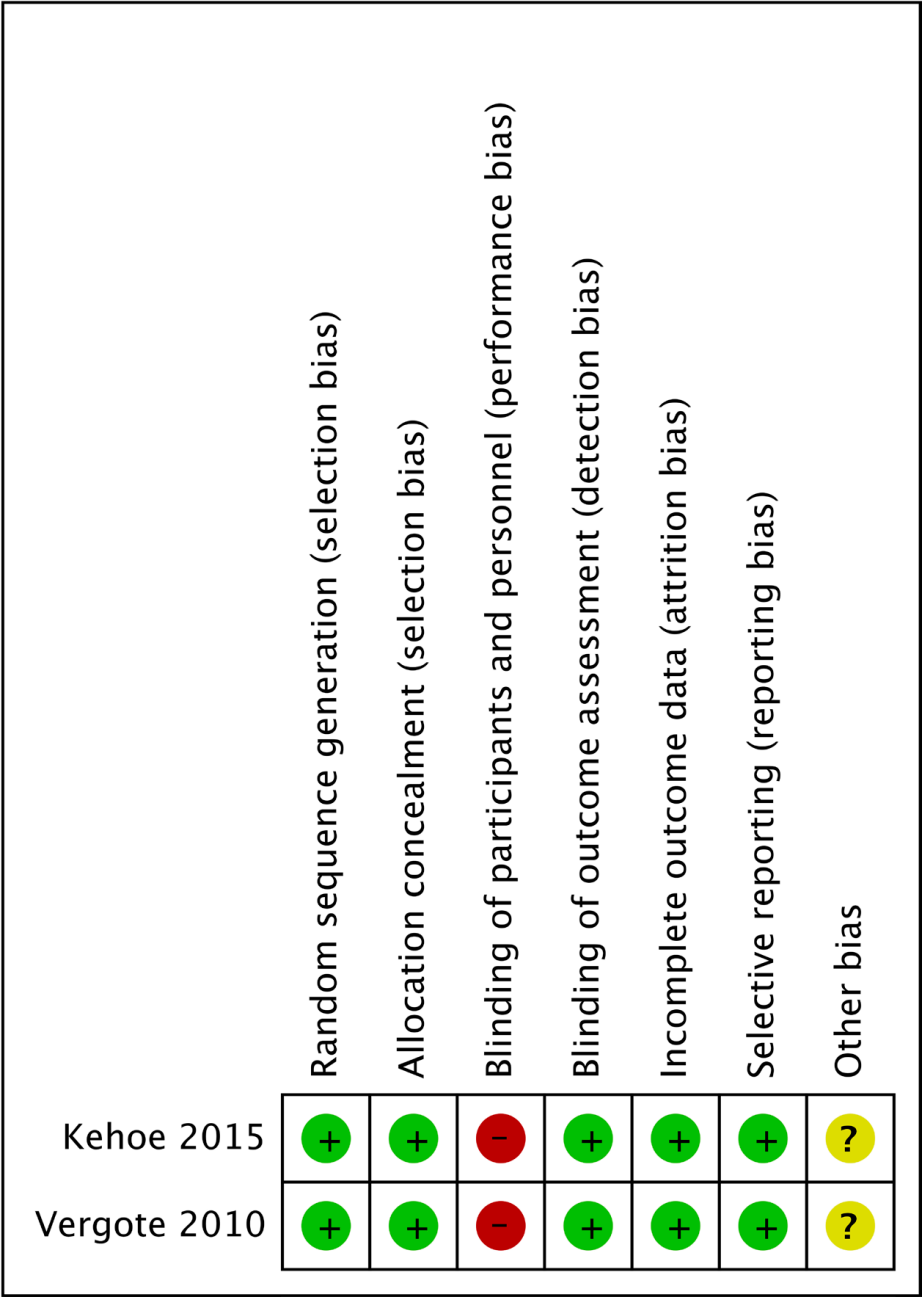
Risk of bias summary for the RCTs

The RCT quality assessment results are shown in Figure 2, which indicates the authors’ judgments regarding the risk of bias for the various categories for each included study. Based on the risk of bias summary, both RCTs had a high risk of performance bias; a low risk of detection bias, attrition bias and reporting bias; and an unclear risk of other bias. There were different risks associated with selection bias pertaining to random sequence generation and allocation concealment. For the observational studies, the quality scores of the retrospective studies are presented in Supplementary Table 1. According to the Newcastle-Ottawa Scale, the 22 included studies were rated as being of good or excellent quality (score range 6–9).

### Meta-analysis

#### Overall survival

**Figure 3:**
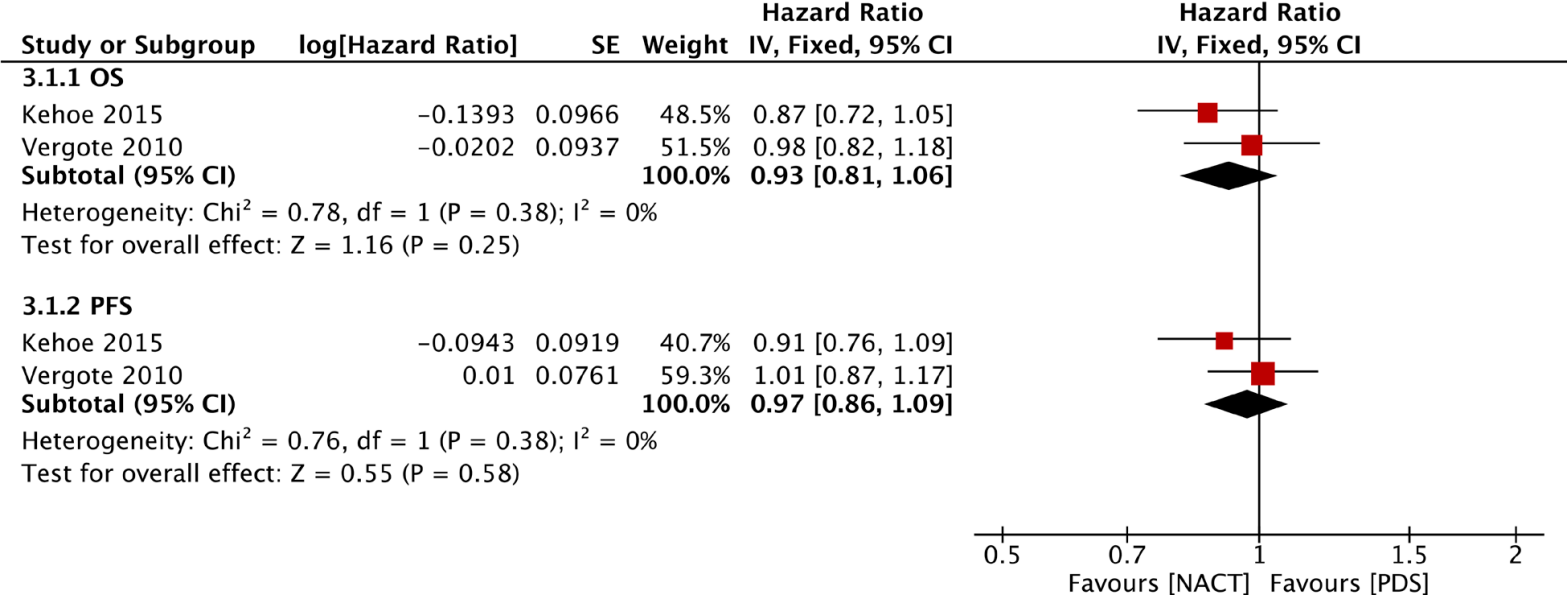
Forest plot of OS and PFS in RCTs

Figure 3 shows the forest plot of OS with NACT-IDS from the RCTs. A fixed-effects model was chosen to evaluate the differences of all included RCTs because of low heterogeneity (*P* = 0.38, I^2^ = 0%). The HR for overall death based on the ITT analysis comparing the NACT group and the PDS group was 0.93 (95% CI: 0.81 to 1.06), which indicated that there was no significant difference between the two groups.

**Figure 4:**
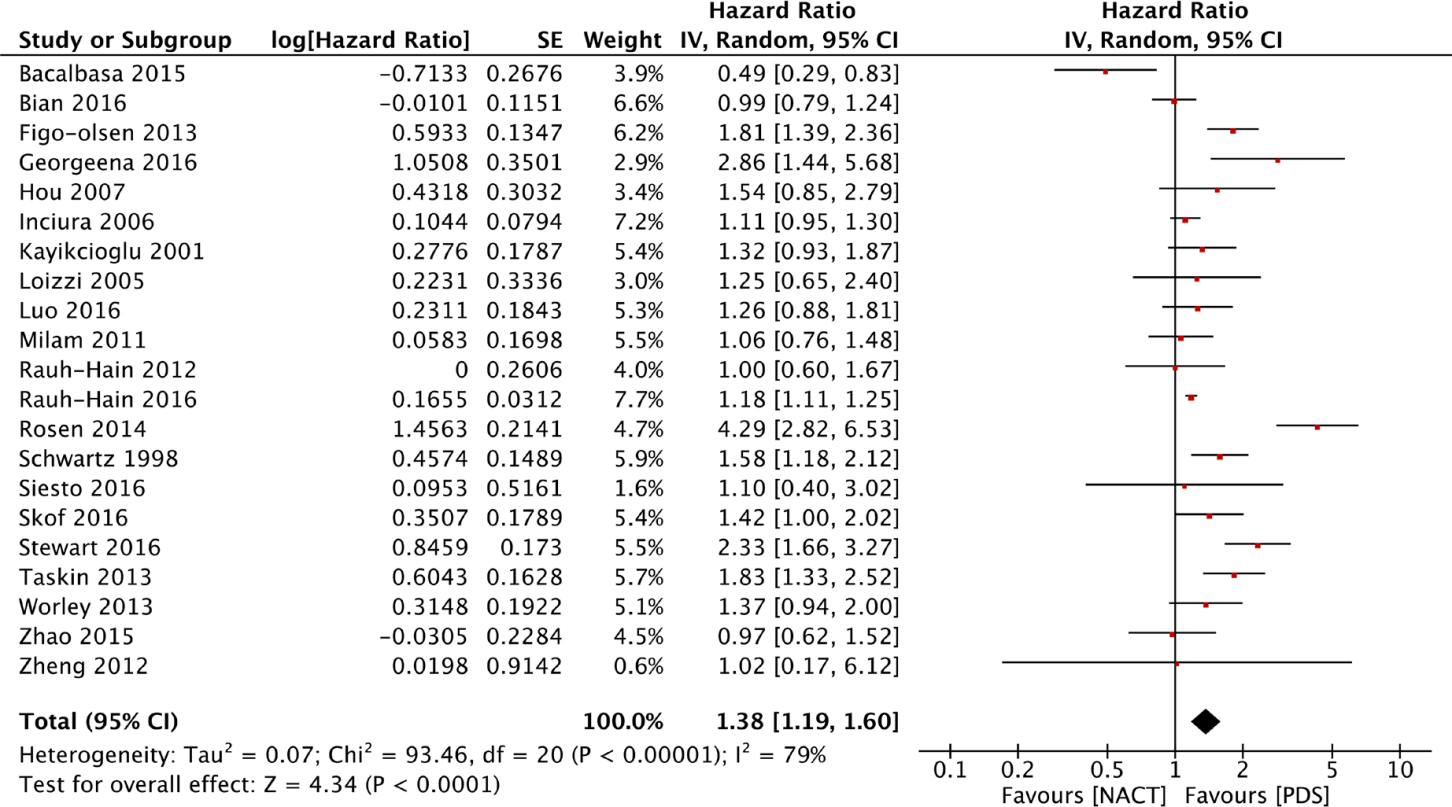
Forest plot of OS in observational studies

The forest plot of OS associated with NACT-IDS from the retrospective studies is presented in Figure 4. A random-effects model was chosen to evaluate the differences among the 21 included retrospective studies because of high heterogeneity (*P* < 0.00001, I^2^ = 77%), and the results indicated that the OS for NACT-IDS is not better than that for PDS (HR = 1.38, 95% CI: 1.19 to 1.60).

#### Progression-free survival

**Figure 5:**
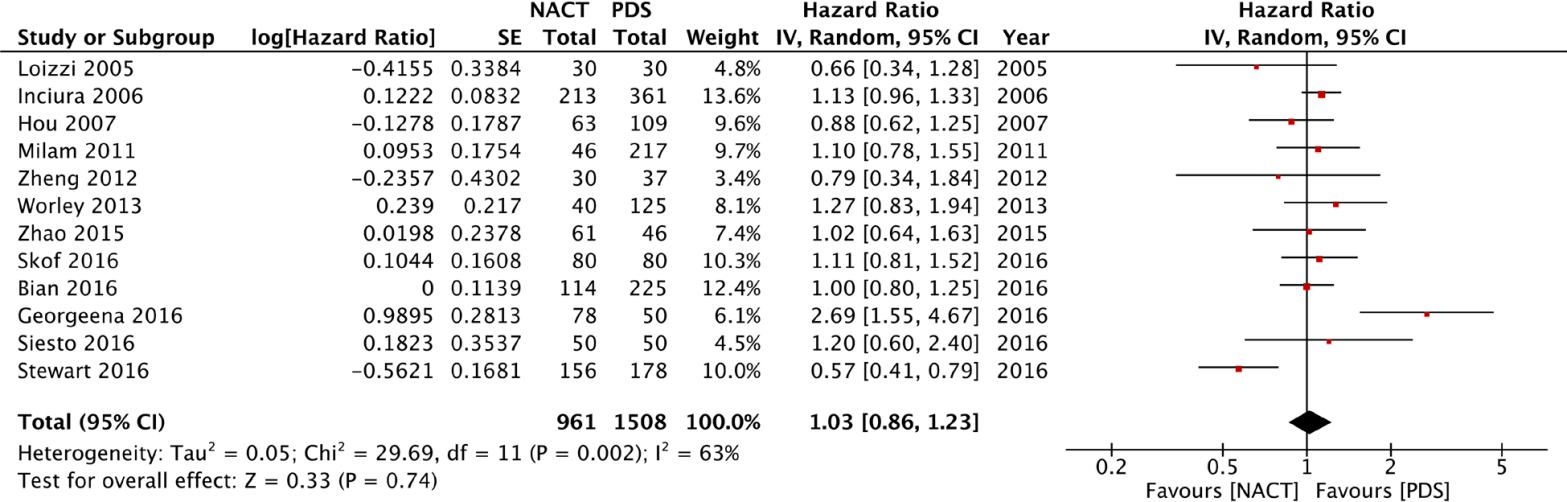
Forest plot of PFS in observational studies

Figure 3 shows the forest plot of PFS associated with NACT-IDS from the two RCTs [12, 13]. The HR for the progression of ovarian cancer based on the ITT analysis comparing the NACT group and the PDS group was 0.97 (95% CI: 0.86 to 1.09). Similarly, the HR of the 12 included retrospective studies was 1.03 (95% CI: 0.86 to 1.23), which is presented in Figure 5. There was no significant difference between the two groups, and all RCTs and observational studies indicated that NACT-IDS does not delay ovarian cancer progression.

#### Adverse events

**Figure 6:**
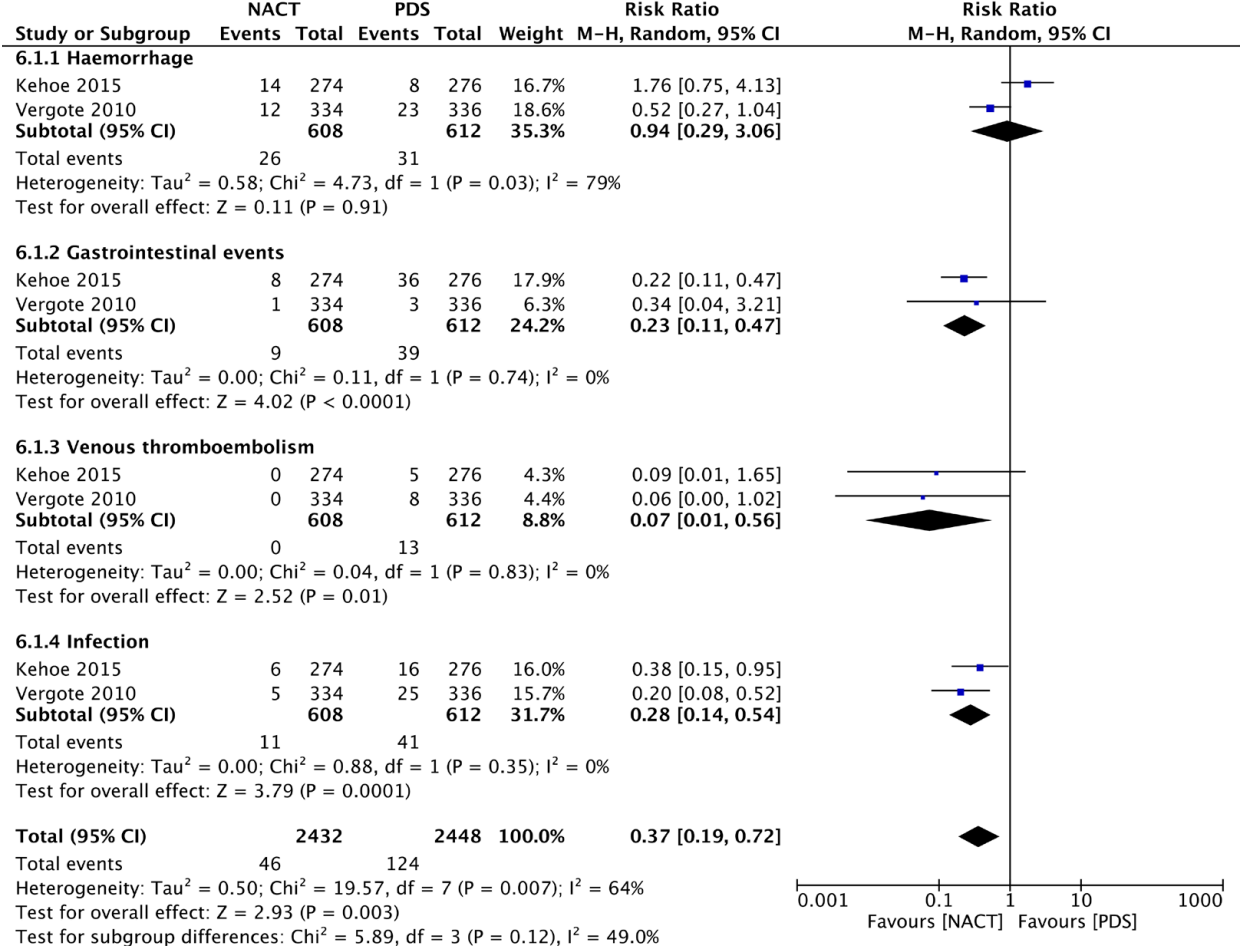
Forest plot of adverse events in RCTs

The adverse events associated with NACT-IDS in the included RCTs are displayed in Figure 6 and mainly include hemorrhage, venous thromboembolism, infection and some gastrointestinal events. Compared with that in the PDS group, the incidence of venous thromboembolism (RR = 0.07, 95% CI: 0.01 to 0.56), infection (RR = 0.28, 95% CI: 0.14 to 0.54), and gastrointestinal events (RR = 0.23, 95% CI: 0.11 to 0.47) was significantly lower in the NACT group. There was no significant difference in the rate of hemorrhage (RR = 0.94, 95% CI: 0.29 to 3.06) between the two groups. There were fewer total adverse events in the NACT group (RR = 0.37, 95% CI: 0.19 to 0.72) than in the PDS group. Therefore, NACT-IDS may reduce the incidence of adverse events, especially venous thromboembolism and infection.

#### Optimal debulking rate

**Figure 7:**
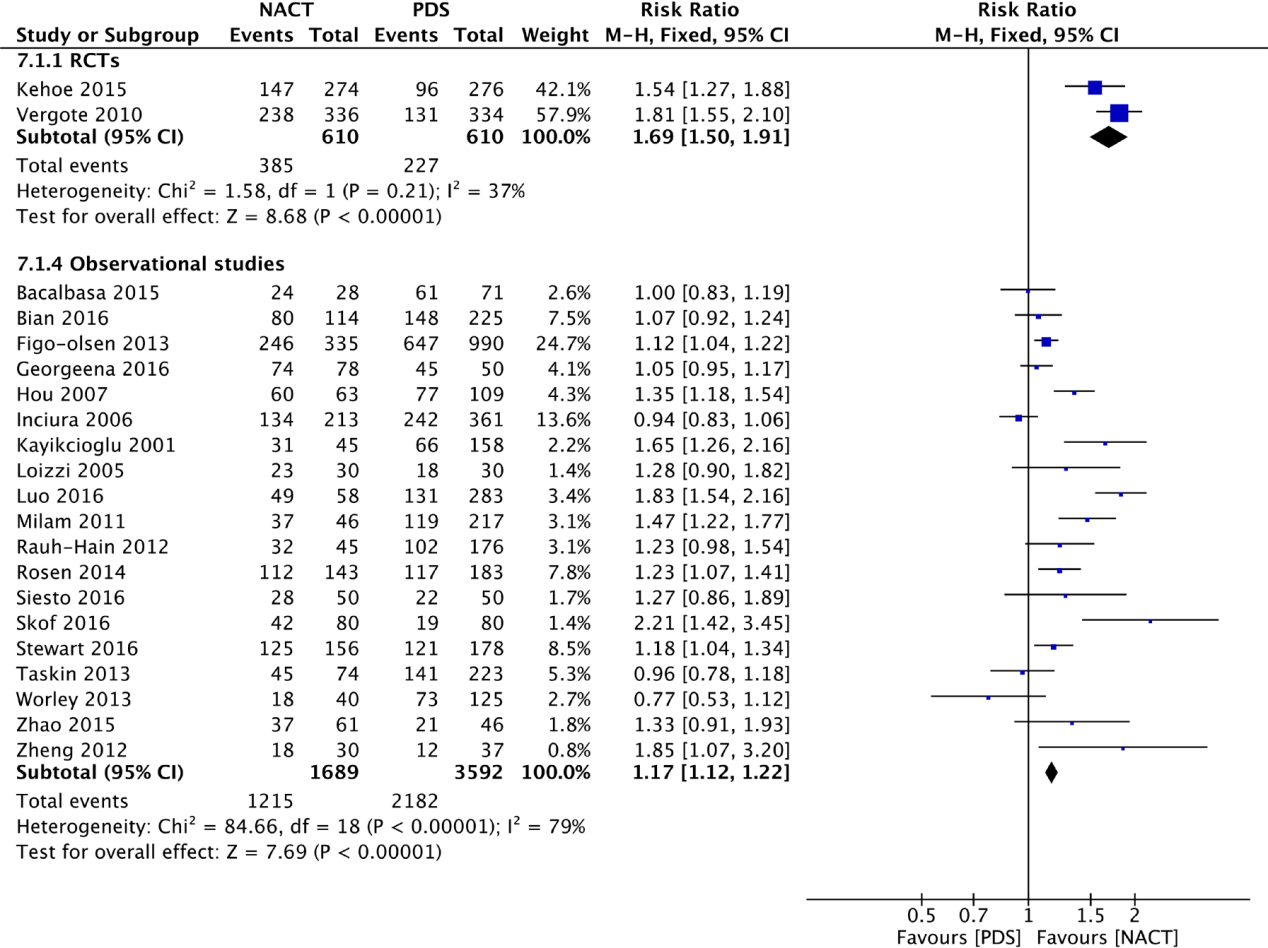
Forest plot of the optimal debulking rate in RCTs and observational studies

The forest plot of the optimal debulking rate based on the two included RCTs and 20 retrospective studies is displayed in Figure 7. The RR for the optimal debulking rate was 1.69 (95% CI: 1.50 to 1.91) in the RCTs and 1.17 (95% CI: 1.12 to 1.22) in the retrospective studies. Therefore, NACT-IDS may be associated with a higher rate of optimal debulking surgery in both RCTs and retrospective studies.

#### Subgroup analysis of OS

**Figure 8:**
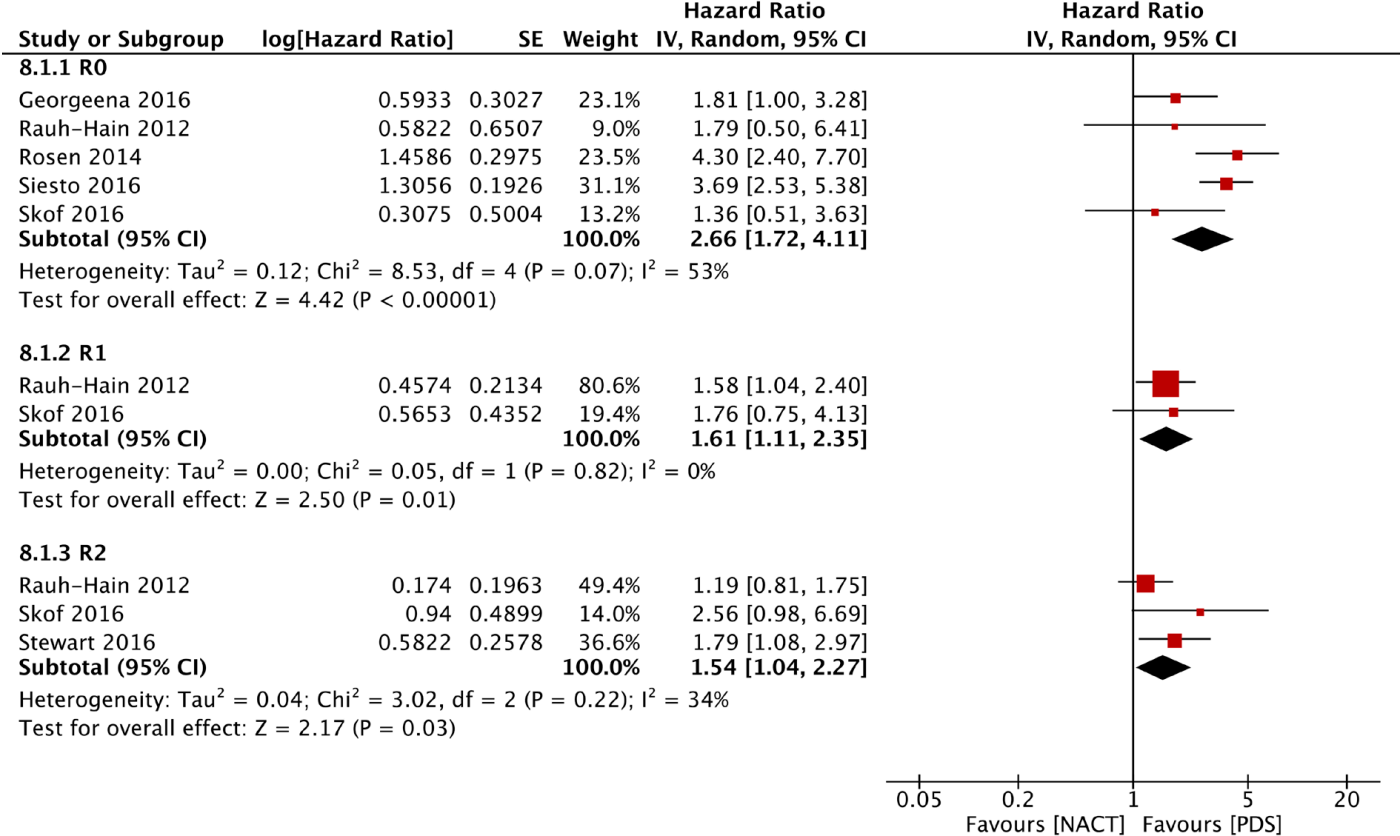
Forest plot of OS of patients with different degrees of residual disease in the observational studies

**Figure 9:**
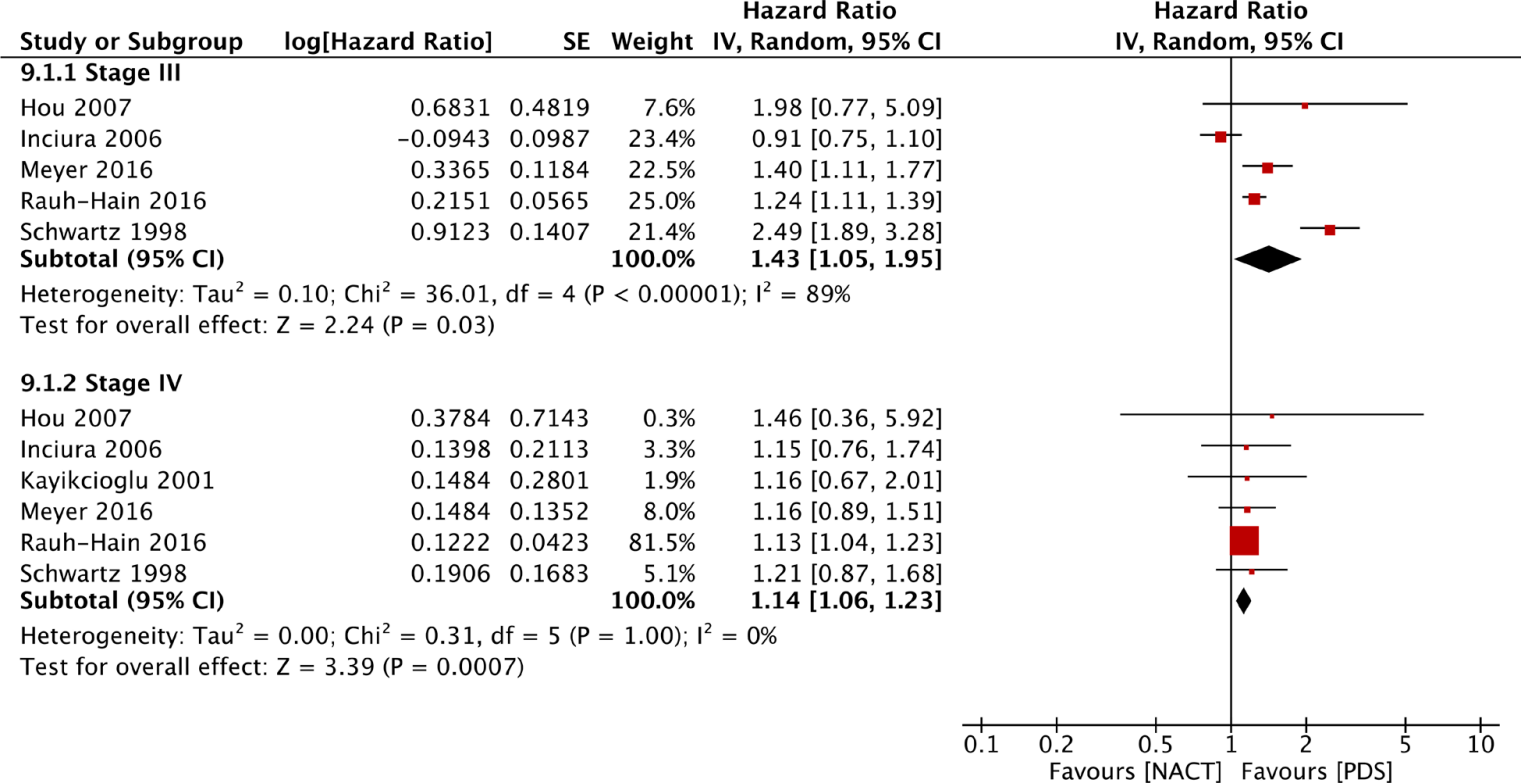
Forest plot of OS of patients with FIGO stage III versus stage IV disease in observational studies

Figure 8 shows that the HR was higher in the NACT group than that for the PDS group in all forest plots (R0: HR=2.66, 95% CI: 1.72 to 4.11; R1: HR=1.61, 95% CI: 1.11 to 2.35; R2: HR=1.54, 95% CI: 1.04 to 2.27), indicating that PDS may contribute to better survival irrespective of the degree of residual disease based on retrospective studies. As shown in Figure 9, PDS also improved the OS of patients with both FIGO stage III (HR = 1.43, 95% CI: 1.05 to 1.95) and stage IV (HR = 1.14, 95% CI: 1.06 to 1.23) disease.

### Sensitivity analysis

Using sensitivity analysis, we excluded each trial at a time and changed the effects model to examine heterogeneity. There was no need to check sensitivity in the RCT group because the heterogeneity for OS was zero and because the number of trials assessing PFS was too small. However, in the retrospective studies group, the pooled HR with high heterogeneity was not significantly affected by any particular study, which demonstrated that the results of our meta-analysis were robust and stable.

### Publication bias

**Figure 10:**
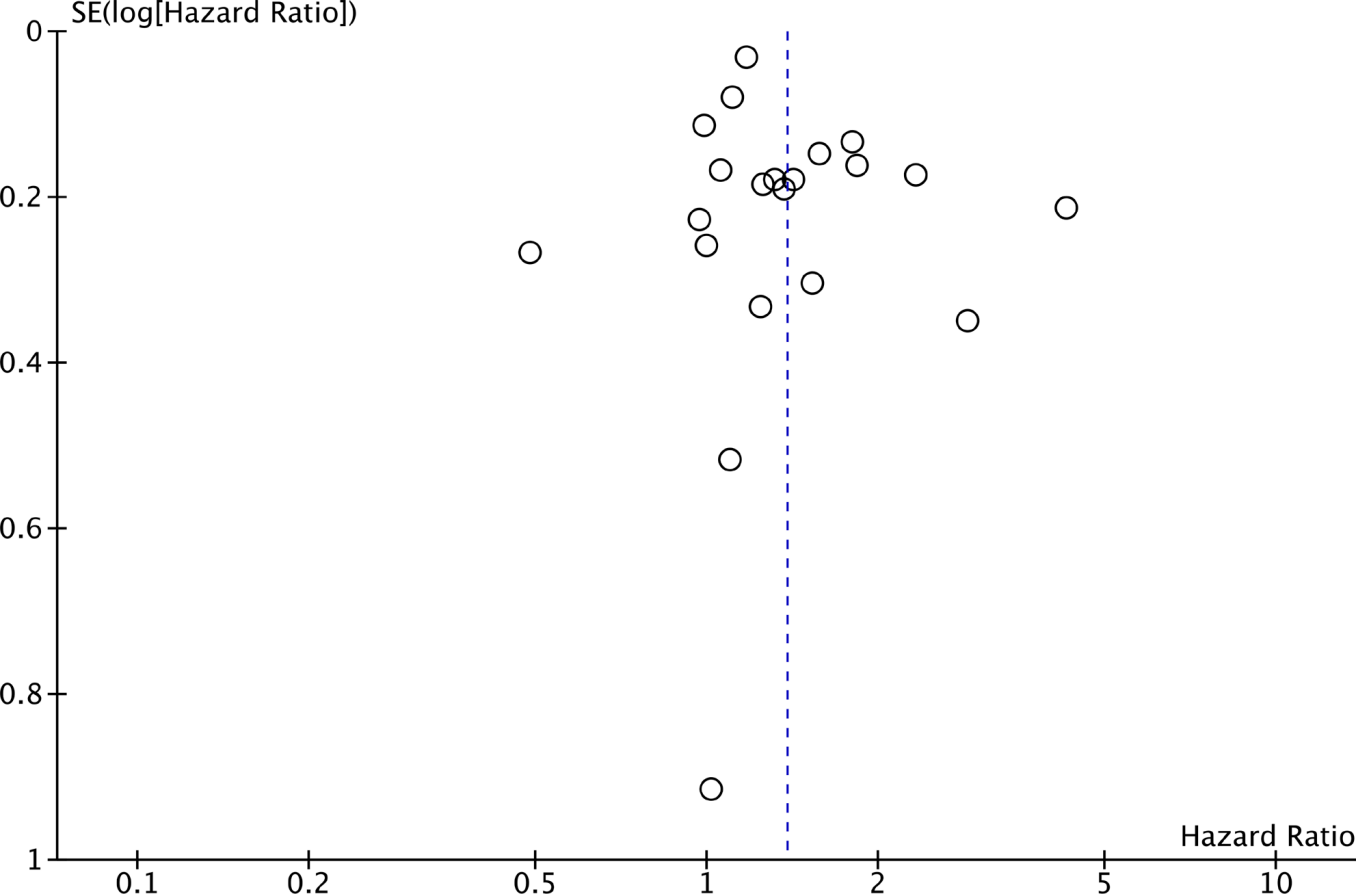
Funnel plot of OS in observational studies

Publication bias of the included trials was evaluated using a funnel plot shown in Figure 10 [58]. The funnel plot of OS based on the 22 included retrospective studies was funnel-shaped and inverted with bilateral symmetry, indicating that there was no publication bias regarding OS. The funnel plot for OS based on RCTs was not generated because the number of RCTs was too small.

## DISCUSSION

Based on the results of our meta-analysis of RCTs, there were no significant differences in the OS and PFS of patients with AOC. NACT-IDS reduced the incidence of surgery-related adverse events, especially venous thromboembolism, infection, and gastrointestinal events. Additionally, debulking surgery in the NACT group resulted in a shorter stay in the intensive care unit and shorter hospital stay. Based on the results of the observational studies, better survival was achieved in the PDS group than that in the NACT group, even though an increased rate of optimal cytoreduction was also observed for the NACT group. Therefore, NACT-IDS is not inferior to PDS-CT in terms of survival outcomes in selected patients.

PDS-CT has been the standard treatment for patients with AOC for a long time [3]. However, since two multicultural, randomized phase III trials (EORTC 55971 [12] and CHORUS [13]) reported that NACT-IDS is not inferior to PDS, the traditional opinion has been challenged. Furthermore, a few updated articles have reported that NACT-IDS does not worsen the prognosis and provides similar survival results to those associated with PDS, although patients receiving NACT typically have more extensive disease [44, 53, 54]. Undoubtedly, NACT-IDS is less invasive than PDS and is associated with an improved optimal cytoreduction rate, quality of life, and perioperative outcomes, such as decreased inpatient hospitalization and decreased blood loss [50, 52, 56].

However, there are some doubts regarding the reduced survival based on the EORTC 55971 and CHORUS trials [19, 59], which reported that optimal cytoreduction did not translate to a survival advantage in the NACT group [37]. As shown in Supplementary Table 1, the median OS was 22.6–24.1 months in the CHORUS trial and 29–30 months in the EORTC 55971 trial. The median OS associated with suboptimal cytoreduction in the Gynecologic Oncology Group (GOG) 111 trial was 24–37 months [60], and it was 49–57 months in the GOG 158 trial in the case of optimal cytoreduction [61]. Although the median OS in the NACT group was longer than that in the PDS group in these two RCTs [12, 13], the survival data were significantly inferior to the survival data in the GOG 111 and GOG 158 trials. The reason underlying the better survival of AOC patients with PDS treatment than that with interval NACT is likely because NACT induced platinum resistance and increased the risk of disease recurrence [62–64]. Additionally, a high proportion of women undergoing NACT had limited performance status with a high disease burden [40].

Some articles analyzed the association of different characteristics of AOC patients with OS. The GOG 182 trial proposed that the survival of patients with more severe preoperative disease is different from that of patients with less severe or moderate disease status, even in the case of R0 resection; this finding indicated that the extent of the disease at diagnosis is the primary determinant of survival and that complex surgery does not affect survival [59]. Meyer 2016 divided patients into FIGO stage III and stage IV groups to compare the survival data and concluded that PDS was associated with increased survival in stage IIIC but not stage IV disease[41]. Van Meurs 2013 proposed that the size of the largest metastatic tumor and clinical stage were associated with treatment benefits. Stage IIIC patients with ≤45 mm metastatic tumors benefited more from PDS, while stage IV patients with > 45 mm metastatic tumors benefited more from NACT [65]. Additionally, Worley 2013 noted that elderly patients undergoing PDS exhibited similar oncologic outcomes as patients undergoing NACT-IDS, indicating that the age of patients was not a determinant of prognosis[48]. Furthermore, the economic assessment involving the use of NACT is important. Administration of NACT treatment to patients in the high-risk subgroup but not in the low-risk subgroup was cost-effective as reported by Poonawalla [66].

Table 1 shows the characteristics of the RCTs [33–36] that were included in the qualitative analysis but excluded from the meta-analysis. These three trials had a total of 441 participants with FIGO stage III or IV disease, namely, 222 in the NACT group and 219 in the PDS group. The chemotherapy regimen was paclitaxel with carboplatin or cisplatin. Preoperative parameters, such as hospital stay and operation time, were shorter in the NACT group. The incidence of postoperative adverse events, including hemorrhage, infection, venous thrombosis and even death, was lower in the NACT group than that in the PDS group. The optimal debulking rate was markedly higher in the NACT group. These three RCTs indicated that NACT-IDS was the superior strategy due to lower surgical aggressiveness and postoperative morbidity and better quality of life scores. Nevertheless, statistical data on the optimal cytoreduction rate and surgery-related events have been published, but survival data are lacking. Completion of patient enrollment and analysis of survival data will further confirm whether PDS or NACT is the superior treatment for women with AOC.

**Table 1:** Characteristics of trials that were included in the qualitative analysis but were excluded from the meta-analysis

Study ID			Onda 2016	Melis 2016	Fagotti 2016
**Design**			RCT	RCT	RCT
**Country**			Japan	Alexandria, U.S. A	Italy
**Recruitment period**			2006/11-2011/10	2011-2013	2011/10-2014/11
**End points**			concerned treatment invasiveness	NR	PFS/perioperative & postoperative outcome① OS/QoL②
**Number of patients**		**NACT**	152	15	55
		**PDS**	149	15	55
		**Total**	301	30	110
**FIGO stage**	**III**	**NACT**	100 (67.1%)	47 (85.5%)	NR
		**PDS**	105 (69.1%)	51 (92.7%)	NR
	**IV**	**NACT**	49 (32.9%)	8 (14.5%)	NR
		**PDS**	47 (30.9%)	4 (7.3%)	NR
**Median age (year)**		**NACT**	60.5	NR	55
		**PDS**	59	NR	54
**Intervention**		**NACT**	NACT (4) +IDS+CT (4)	NACT (3) +IDS+CT (3)	NACT (3/4) +IDS
		**PDS**	PDS+ACT (8)	PDS+ACT (6)	PDS+ACT (6)
		**CR**	TC	TP	TP
**Median OS (month)**		**NACT**	NR	NR	NR
		**PDS**	NR	NR	NR
**Median PFS (month)**		**NACT**	NR	NR	NR
		**PDS**	NR	NR	NR
**Optimal debulking rate**		**NACT**	NR	86.70%	NR
		**PDS**	NR	6.70%	NR
**Hospital stay (day)**		**NACT**	NR	86.7% <3d	6
		**PDS**	NR	100% >3d	12
**Operation time (min)**		**NACT**	273	93.3% <120 min	275
		**PDS**	341	80% >120 min	451
**Major** **post-surgical complications**	**Hemorrhage** **(g/dl)**	**NACT**	NR	1.4	80% <1g/dl
		**PDS**	NR	3.25	73.3% >1g/dl
	**Infection**	**NACT**	0.8%	NR	NR
		**PDS**	0.7%	NR	NR
	**Venous thrombosis**	**NACT**	3.1%	0	NR
		**PDS**	4.8%	5.5%	NR
	**Death**	**NACT**	0	0	NR
		**PDS**	0.7%	5.5%	NR

(Abbreviations: RCT= randomized controlled trial; NACT=neoadjuvant chemotherapy; PDS=primary debulking surgery; ACT=adjuvant chemotherapy; OS=overall survival; PFS=progression-free survival; QoL=Quality of life; TP=cisplatin and paclitaxel; TC= carboplatin and paclitaxel; CR=chemotherapy regimen; NR=not reported.).

Five similar meta-analyses have been conducted prior to our study [18, 67–70]. The meta-analysis by Bristow [18] included 835 patients and 22 cohort studies, all using the traditional definition of NACT, and reported that NACT was associated with inferior OS than was primary surgery. Kang [67] included twenty-one studies of various types with different definitions of NACT and indicated that NACT increased the rate of optimal debulking surgery by gynecologic oncologists. Ma [68] analyzed only two trials (Rose [10] and Vergote [71]) and concluded that there was no significant difference in the median OS and PFS between the two studied groups. The meta-analysis by Tangjitgamol [69] included three trials and assessed the effectiveness and complications associated with IDS, based on the traditional definition, in women with AOC. Another new meta-analysis by Zeng[70], using different definitions of NACT, indicated the lack of significant differences in OS and PFS between the groups and concluded that NACT was a favorable treatment option due to its higher optimal debulking rate, despite our belief that there may have been some incorrect data extraction.

Our study has several strengths and innovations. First, none of the previously published meta-analyses had differentiated between the different definitions of NACT and IDS, even though the previous definition of NACT has been abandoned. Only the current definition of NACT-IDS was discussed in our article; thus, the results are more convincing. Second, this comprehensive meta-analysis included updated articles and presented a new group of recent studies. Different types of study designs were included in our article. Observational studies and RCTs were classified into separate groups. These two types of studies were separately analyzed to yield independent results, which were then statistically evaluated. Third, all extracted data were included in an ITT analysis, and the total number of participants in the NACT group was used to extract the HR values. Therefore, the percentage of patients who underwent NACT but not IDS due to the lack of response had no effect on the overall and subgroup survival outcomes.

Our meta-analysis has several limitations. First, as with any meta-analysis and systematic review, the conclusions drawn from the data are subject to the limitations of the included original articles themselves. The two different types of study designs have their own strengths and weaknesses that affect the interpretation and results. Second, in the RCT group, only two moderate-sized RCTs were analyzed, and the risk of bias was intermediate, although we tried to control for bias, especially for trials conducted before 2010. Regarding the observational studies group, the most important limitation of the current study is the high degree of heterogeneity among the included 22 retrospective studies. Even though a random-effects model was used to allow for considerable variance among studies, the heterogeneity may have led to unconvincing results in our meta-analysis. Third, the HR with CIs of OS and PFS was the best parameter for the estimation of the effect size. However, these data were not presented in all the original articles and thus had to be estimated from Kaplan-Meier survival curves, and admittedly, this approach has limitations. Fourth, the limitations regarding ethnicity are associated with a potential selection bias, which could have affected the results of this study [72]. Finally, the regimens and cycles of chemotherapy differed among the included trials, which may have affected the survival data. However, none of these factors had any significant effect on survival outcomes in the reported studies.

## CONCLUSIONS

In conclusion, NACT-IDS is associated with better perioperative outcomes and an improved optimal cytoreduction rate, while it does not improve OS. NACT-IDS is not inferior to PDS-CT in terms of survival outcomes in selected patients with AOC. Our consistent objective when performing debulking surgery is to resect all macroscopic tumors to eliminate residual disease. Therefore, we need to confirm the role of NACT-IDS and focus on candidate selection for NACT in more multicenter RCTs with different and larger populations and deeper analyses. We look forward to obtaining the end point of the survival data from the previously mentioned ongoing RCTs [33–35].

## SUPPLEMENTARY MATERIALS TABLE




