# Supplementary material for: The role of neoadjuvant chemotherapy followed by interval debulking surgery in advanced ovarian cancer: a systematic review and meta-analysis of randomized controlled trials and observational studies
Source: Oncotarget. 2017 Dec 27;9(9):8614–28. doi: 10.18632/oncotarget.23808 (PMC5823572; doi:10.18632/oncotarget.23808)
Supplement: Supplementary file 1 [file oncotarget-09-8614-s001.pdf]

The role of neoadjuvant chemotherapy followed by interval debulking surgery in advanced ovarian cancer: a systematic review and meta-analysis of randomized controlled trials and observational studies

SUPPLEMENTARY MATERIALS

Supplementary Table 1: Characteristics of included trials in the meta-analysis. See_Supplementary_Table 1
