# Supplementary material for: The role of neoadjuvant chemotherapy followed by interval debulking surgery in advanced ovarian cancer: a systematic review and meta-analysis of randomized controlled trials and observational studies
Source: Oncotarget. 2017 Dec 27;9(9):8614–28. doi: 10.18632/oncotarget.23808 (PMC5823572; doi:10.18632/oncotarget.23808)
Supplement: Supplementary file 2 [file oncotarget-09-8614-s002.docx]

| **Study ID** | **Design** | **Country** | **Recruitment period** | **FIGO Stage** | **Chemotherapy Regimen** | **Number of patients** | | | | **Median age (years)** | | | | **Median OS (months)** | | | | **Median PFS (months)** | | | | **Optimal debulking rate** | | | | **Study quality** |
| --- | --- | --- | --- | --- | --- | --- | --- | --- | --- | --- | --- | --- | --- | --- | --- | --- | --- | --- | --- | --- | --- | --- | --- | --- | --- | --- |
|  |  |  |  |  |  | **NACT** | **PDS** | **Total** | **NACT**  **+IDS** | **NACT** | | **PDS** | | **NACT** | | **PDS** | | **NACT** | | **PDS** | | **NACT** | | **PDS** | |  |
| **RCTs** | | | | | | | | | | | | | | | | | | | | | | | | | |  |
| Kehoe 2015 | RCT | U.K. | 2004-2010 | III/IV | TC | 274 | 276 | 550 | 20.80% | | 65 | | 66 | | 24.1 | | 22.6 | | 12 | | 10.7 | | 53.65% | | 34.78% | - |
| Vergote 2010 | RCT | Europe | 1998-2006 | IIIC/IV | TP | 334 | 336 | 670 | 11.68% | | 63 | | 62 | | 30 | | 29 | | 12 | | 12 | | 70.83% | | 39.22% | - |
| **Observational studies** | | | | | | | | | | | | | | | | | | | | | | | | | | |
| Stewart 2016 | RCA | Canada | 2003-2011 | III-IV | NR | 156 | 178 | 334 | 0.00% | | 60.0 | | 56.0 | | 64.0 | | 30.7 | | NR | | NR | | 80.00% | | 68.00% | 7 |
| Skof 2016 | RCA | Slovenia | 2005-2007 | IIIC | TC | 80 | 80 | 160 | 0.00% | | 64.8 | | 60.2 | | 24.8 | | 31.6 | | 14.1 | | 17.7 | | 52.00% | | 24.00% | 7 |
| Siesto 2016 | RCA | Italy | 2009-2016 | IIIC-IV | TC | 50 | 50 | 100 | 0.00% | | 63.2 | | 60.8 | | 44.5 | | 43.2 | | 27.7 | | 23.0 | | 56.00% | | 44.00% | 7 |
| Rauh-Hain 2016 | RCA | USA | 2003-2011 | IIIC-IV | NR | 2935 | 2935 | 5870 | 26.00% | | 56.0 | | 56.0 | | 32.1 | | 37.3 | | NR | | NR | | NR | | NR | 9 |
| Meyer 2016 | RCA | USA | 2003-2012 | IIIC-IV | TP | 372 | 786 | 1158 | 0.00% | | NR | | NR | | NR | | NR | | NR | | NR | | NR | | NR | 9 |
| Luo 2016 | RCA | Korea | 1990-2010 | IIIC-IV | TP | 58 | 283 | 341 | 0.00% | | 41.0 | | 51.0 | | 41.0 | | 51.0 | | NR | | NR | | 84.50% | | 46.30% | 8 |
| Georgeena 2016 | RCA | India | 2008-2011 | IIIC-IV | TC | 78 | 50 | 128 | 0.00% | | NR | | NR | | 44.0 | | 58.0 | | 39.0 | | 56.0 | | 94.80% | | 90.00% | 6 |
| Bian 2016 | RCA | China | 2005-2010 | IIIC-IV | TC | 114 | 225 | 339 | 0.00% | | 53.0 | | 50.7 | | 25.0 | | 25.0 | | 11.0 | | 10.0 | | 70.20% | | 65.80% | 8 |
| Zhao 2015 | RCA | China | 2004-2010 | III-IV | TC/TP/PC/PAC | 61 | 46 | 107 | 0.00% | | 56.0 | | 57.4 | | 42.0 | | 55.0 | | 16.0 | | 17.0 | | 60.66% | | 45.65% | 8 |
| Bacalbasa 2015 | RCA | Romania | 2002-2014 | IIIC-IV | TP | 71 | 28 | 99 | 9.86% | | 54.0 | | 54.0 | | 43.0 | | 33.0 | | NR | | NR | | 85.90% | | 85.60% | 6 |
| Rosen 2014 | RCA | Canada | 2001-2011 | IIIC-IV | TP | 143 | 183 | 326 | 0.00% | | 61.6 | | 56.7 | | 41.0 | | NR | | NR | | NR | | 78.10% | | 63.90% | 6 |
| Worley 2013 | RCA | USA | 2000-2010 | IIIC-IV | TC | 40 | 125 | 165 | 0.00% | | 74.0 | | 75.0 | | 29.0 | | 33.0 | | 17.0 | | 15.0 | | 45.00% | | 58.40% | 8 |
| Taskin 2013 | RCA | Turkey | 2001-2010 | IIIC-IV | TC | 74 | 223 | 297 | 0.00% | | 60.5 | | 56.4 | | 40.0 | | 64.0 | | NR | | NR | | 60.80% | | 63.20% | 8 |
| Figo-olsen 2013 | RCA | Denmark | 2005-2011 | IIIC-IV | TP | 515 | 990 | 1505 | 34.95% | | 65.0 | | 65.0 | | 29.4 | | 31.9 | | NR | | NR | | 74.00% | | 66.00% | 8 |
| Zheng 2012 | RCA | China | 2006-2009 | IIIC-IV | TC/PAC | 30 | 37 | 67 | 0.00% | | 55.8 | | 54.5 | | 41.2 | | 39.1 | | 27.1 | | 24.3 | | 60.00% | | 32.40% | 8 |
| Rauh-Hain 2012 | RCA | USA | 1995-2007 | IV | TP | 66 | 176 | 242 | 31.82% | | 62.0 | | 62.0 | | 33.0 | | 29.0 | | 14.0 | | 11.0 | | 71.00% | | 58.00% | 7 |
| Milam 2011 | RCA | USA | 1993-2005 | IIIC-IV | TC/TP | 46 | 217 | 263 | 0.00% | | 61.0 | | 57.0 | | 43.2 | | 39.6 | | 18.0 | | 14.4 | | 80.00% | | 55.00% | 9 |
| Hou 2007 | RCA | USA | 1998-2005 | IV | TC | 63 | 109 | 172 | 0.00% | | 64.1 | | 62.7 | | 46.0 | | 47.0 | | 16.0 | | 14.0 | | 95.00% | | 71.00% | 7 |
| Inciura 2006 | RCA | Lithuania | 1993-2000 | III-IV | PC | 213 | 361 | 574 | 0.00% | | NR | | NR | | 23.7 | | 25.4 | | 13.3 | | 15.0 | | 63.00% | | 67.00% | 8 |
| Loizzi 2005 | RCS | Italy | 1994-2003 | IIIC-IV | TC | 30 | 30 | 60 | 16.67% | | 64.0 | | 58.0 | | 32.0 | | 40.0 | | 21.0 | | 16.0 | | 76.00% | | 60.00% | 7 |
| Kayikcioglu 2001 | RCA | Turkey | 1991-2000 | IIIC-IV | PC/TC | 45 | 158 | 203 | 0.00% | | 58.5 | | 53.6 | | 34.1 | | 37.9 | | 16.3 | | 13.9 | | 68.90% | | 41.70% | 8 |
| Schwartz 1998 | RCA | USA | 1979-1996 | IIIC-IV | TP | 59 | 206 | 265 | 30.51% | | 67.0 | | 60.0 | | 12.8 | | 26.2 | | NR | | NR | | NR | | NR | 7 |

**Table 1. Characteristics of included trials in the meta-analysis.** (Abbreviations: RCT=randomized controlled trial; RCA=retrospective cohort analysis; RCS=retrospective case-control study; NACT=neoadjuvant chemotherapy; PDS=primary debulking surgery; ACT=adjuvant chemotherapy; NACT+IDS=percentage of patients who had undergone NACT followed by IDS, among all patients who underwent NACT irrespective of IDS; OS=overall survival; PFS=progression-free survival; TP=cisplatin and paclitaxel; TC=carboplatin and paclitaxel; PC=cisplatin and cyclophosphamide; PAC=cisplatin and epirubicin cyclophosphamide; NR=not reported.)
